# Normalization of prostate specific antigen in patients treated with intensity modulated radiotherapy for clinically localized prostate cancer

**DOI:** 10.1186/1748-717X-5-80

**Published:** 2010-09-16

**Authors:** Matthew D Schmitz, Gilbert DA Padula, Patrick Y Chun, Alan T Davis

**Affiliations:** 1 College of Human Medicine, Michigan State University, East Lansing, MI, USA; 2 Department of Medicine, Michigan State University College of Human Medicine, East Lansing, Michigan, USA; 3 Lacks Cancer Center, Saint Mary's Health Care, Grand Rapids, Michigan, USA; 4 Department of Surgery, Michigan State University, Grand Rapids, MI, USA; 5 Department of Research, Grand Rapids Medical Education Partners, Grand Rapids, MI, USA

## Abstract

**Background:**

The purpose of this study was to determine the expected time to prostate specific antigen (PSA) normalization with or without neoadjuvant androgen deprivation (NAAD) therapy after treatment with intensity modulated radiotherapy (IMRT) for patients with clinically localized prostate cancer.

**Methods:**

A retrospective cohort research design was used. A total of 133 patients with clinical stage T1c to T3b prostate cancer (2002 AJCC staging) treated in a community setting between January 2002 and July 2005 were reviewed for time to PSA normalization using 1 ng/mL and 2 ng/mL as criteria. All patients received IMRT as part of their management. Times to PSA normalization were calculated using the Kaplan-Meier method. Significance was assessed at p < 0.05.

**Results:**

Fifty-six of the 133 patients received NAAD (42.1%). Thirty-one patients (23.8%) received radiation to a limited pelvic field followed by an IMRT boost, while 99 patients received IMRT alone (76.2%). The times to serum PSA normalization < 2 ng/mL when treated with or without NAAD were 298 ± 24 and 302 ± 33 days (mean ± SEM), respectively (p > 0.05), and 303 ± 24 and 405 ± 46 days, respectively, for PSA < 1 ng/mL (p < 0.05). Stage T1 and T2 tumors had significantly increased time to PSA normalization < 1 ng/mL in comparison to Stage T3 tumors. Also, higher Gleason scores were significantly correlated with a faster time to PSA normalization < 1 ng/mL.

**Conclusions:**

Use of NAAD in conjunction with IMRT leads to a significantly shortened time to normalization of serum PSA < 1 ng/mL in patients with clinically localized prostate cancer.

## Background

Prostate cancer is a prominent cause of morbidity and mortality among men. In 2009, over 190,000 men were diagnosed and over 25,000 died of this disease in the United States alone [1]. The utilization of prostate specific antigen (PSA) screening has resulted in improved detection of this malignancy in its early stages. Current management options for localized prostate cancer include radical prostatectomy, external beam radiation therapy, brachytherapy, and active surveillance. Both external beam radiation therapy (EBRT) and brachytherapy may be combined with neoadjuvant androgen deprivation therapy (NAAD). While there is much debate about which modality provides the optimal treatment for localized disease, radiation therapy with or without the use of NAAD has been a mainstay of prostate cancer management for many years and has been studied extensively.

Intensity modulated radiation therapy (IMRT) is a sophisticated version of 3-dimensional conformal radiation therapy (3D-CRT) which has become widely used in the United States [2-4]. The ability of IMRT to vary the intensity of its radiation beam allows for more precise dose distribution around complex target volumes. This makes it especially amenable to the treatment of localized prostate cancer. IMRT has been shown to allow for significantly higher doses to the prostate itself, while simultaneously decreasing the radiation dose to surrounding normal tissues [5].

A number of studies have shown improvement in outcome for patients with locally advanced prostate cancer treated with both external beam radiotherapy and NAAD [6,7]. However, there is little in the peer-reviewed literature describing the effect upon PSA normalization of using NAAD in conjunction with IMRT in prostate cancer. The purpose of this study is to determine the expected time to PSA normalization with or without NAAD after treatment with IMRT for patients with clinically localized prostate cancer.

## Methods

Between January 2002 and July 2005, 133 patients with clinical stage T1c to T3b prostate cancer (using the 2002 AJCC staging system) treated in a community setting were reviewed for time to PSA normalization. All patients received IMRT as part of their management. Thirty-one patients (23.5%) received radiation to a limited pelvic field followed by an IMRT boost, while 99 patients (76.5%) received IMRT alone. Three patients' boost statuses were not recorded. Treatment planning was accomplished through the use of multi-slice computerized tomography (CT) scanning of the prostate. The median prescribed dose to the prostate was 75.6 Gy given in 1.8 Gy fractions.

For patients that underwent pelvic radiotherapy, a standard four field box technique was utilized. Simulation took place with the utilization of a customized immobilization device. 6 or 15 MV photons were utilized. After a dose of 45-50.4 Gy, patients underwent an IMRT boost. The IMRT technique was the same regardless of whether patients received IMRT alone or following pelvic radiotherapy. IMRT took place with the utilization of a customized immobilization device. 6MV photons were used. Dose was prescribed to the planning target volume (PTV). The PTV was prescribed at 1.0 cm around the prostate and seminal vesicles and at 0.6 cm around rectoprostatic interface.

Fifty-six patients received NAAD therapy in conjunction with IMRT and 77 received IMRT alone. NAAD therapy consisted of Lupron (leuprolide) and Casodex (bicalutamide) for a median treatment length of six months (range 1 - 26 months). NAAD was given two months prior and concurrent to radiotherapy as has been the practice in our clinic.

Serum prostate specific antigen levels were collected prior to either NAAD therapy or radiation therapy. The mean pretreatment serum PSA level was 9.5 ± 0.6 (mean ± standard error of the mean [SEM]) ng/mL. All 133 patients had a pre-treatment PSA level drawn as well as at least one post-treatment PSA level. Patients who had a pre-treatment PSA level of less than 2 ng/mL were not included in the 133 patients as they were already below the decided upon standard for PSA normalization. Time was initiated from the start of radiotherapy and normalization was assessed during the follow up period. Patients were followed at months one, four, and every three months thereafter for the first two years of the follow up period. Then, patients were assessed every six months between years two and five post radiotherapy. At each follow up, patients also underwent a digital rectal examination. PSA levels were considered to have normalized at either a PSA of 2 ng/mL or 1 ng/mL post-treatment. These levels were chosen as these were the standard cutoffs used in our clinic for time to PSA normalization.

The quantitative data are expressed as the mean ± SEM. Ordinal data are expressed as the median, with the range in parentheses. Times to PSA normalization were calculated using the Kaplan-Meier method. Differences between means for quantitative variables for two or three treatments were analyzed using the two-tailed t-test or the one way ANOVA, respectively. Associations between independent variables were performed using Pearson's correlation coefficient. The relationship between Gleason score and time to normalization was tested using the Spearman correlation coefficient. Significance was assessed at p < 0.05.

Multivariate analyses utilizing the Cox Proportional Hazards Model were performed on various hypothesized predictors of normalization of PSA to below both 2 ng/mL and 1 ng/mL. Any variable which had a significance level < 0.2 in the univariate analysis was tested in the multivariate model. Only variables which tested to be significant in the regression analysis (p < 0.05) were included in the final equation.

## Results

Table 1 describes the demographic and clinical data for the subjects. Data for the subjects, related to the time of normalization of the PSA to less than either 1 ng/mL or 2 ng/mL and related information are on Tables 2 and 3, respectively. PSA levels were normalized to below 1 ng/mL in 93 of 133 total patients. Those patients treated with IMRT plus NAAD had a significantly shorter time to normalization, relative to the IMRT alone group using PSA normalization to below 1 ng/mL as an endpoint. PSA levels were normalized to below 2 ng/mL in 119 of the 133 total patients in the follow-up period. Information on NAAD was available for 80 of these patients. Those patients treated with IMRT plus NAAD showed a similar time to normalization, relative to the use of IMRT alone, with respect to PSA normalization to below 2 ng/mL as an endpoint.

**Table 1:** Patient demographic and clinical data

Variable	Value
Age (n = 121)*	69.5 ± 0.7
NAAD	56/83 (67.5%)
Time of hormonal treatment (mon; n = 52)*	9.3 ± 1.1
Gleason Score (n = 132)^†^	6 (5 - 10)
Pre-PSA (ng/mL)*	9.5 ± 0.6
Unilateral disease	65/117 (55.6%)
IMRT Boost	31/130 (23.9%)
Tumor stage	
T1c	100/130 (76.9%)
T2	5/130 (3.9%)
T2a	13/130 (10.0%)
T2b	4/130 (3.1%)
T2c	2/130 (1.5%)
T3	3/130 (2.3%)
T3a	2/130 (1.5%)
T3b	1/130 (0.8%)

Abbreviation: NAAD = Neoadjuvant androgen deprivation therapy; PSA = Prostate specific antigen; IMRT = intensity modulated radiation therapy

* mean ± SEM

† median (range in parentheses)

**Table 2:** Data for subjects related to the time of normalization of PSA < 1 ng/mL

	Normalization of PSA to < 1 ng/mL
Time to normalization (d) * (n = 93)	332 ± 18
IMRT with or without NAAD*	
With NAAD (n = 49)	303 ± 24^‡^
Without NAAD (n = 21)	405 ± 46^‡^
IMRT Boost*	
Yes (n = 27)	359 ± 34
No (n = 66)	328 ± 22
Tumor Stage*^,§^	
T1c (n = 70)	329 ± 18 a
T2, T2a, T2b, T2c (n = 16)	440 ± 59 a
T3, T3a, T3b (n = 6)	154 ± 33 b
Correlation to time of normalization of PSA^†^	
Age at time of CT (n = 84)	0.01
Length of hormone trt (n = 45)	0.19
Gleason Score (n = 93)	-0.22^‡^
Pre-PSA (n = 93)	-0.13

Abbreviation: PSA = Prostate specific antigen; NAAD = Neoadjuvant androgen deprivation therapy; IMRT = intensity modulated radiation therapy

* mean ± SEM

† correlation coefficient

‡ p < 0.05

§ values in a column followed by a different letter are significantly different (p < 0.05)

**Table 3:** Data for subjects related to the time of normalization of PSA < 2 ng/mL

	Normalization of PSA to < 2 ng/mL
Time to normalization (d) * (n = 119)	289 ± 15
IMRT with or without NAAD*	
With NAAD (n = 54)	298 ± 24
Without NAAD (n = 26)	303 ± 33
IMRT Boost*	
Yes (n = 28)	308 ± 25
No (n = 91)	283 ± 18
Tumor Stage*^,§^	
T1c (n = 90)	280 ± 15 b
T2, T2a, T2b, T2c (n = 21)	375 ± 42 a
T3, T3a, T3b (n = 6)	154 ± 33 c
Correlation to time of normalization of PSA^†^	
Age at time of CT (n = 108)	-0.12
Length of hormone trt (n = 50)	0.22
Gleason Score (n = 119)	-0.13
Pre-PSA (n = 119)	-0.17

Abbreviation: PSA = Prostate specific antigen; NAAD = Neoadjuvant androgen deprivation therapy; IMRT = intensity modulated radiation therapy

* mean ± SEM

† correlation coefficient

‡ p < 0.05

§ values in a column followed by a different letter are significantly different (p < 0.05)

Time to normalization of PSA below 1 ng/mL was assessed as a function of tumor stage (Table 2). Subjects with stage T2 tumors had the longest time to normalization, followed by subjects with T1 and T3 tumors, respectively. The time to normalization for the subjects with T3 tumors was significantly less than for subjects with the other tumor types.

Time to normalization of PSA below 2 ng/mL was assessed as a function of tumor stage (Table 3). Subjects with stage T2 tumors had the longest time to normalization, followed by subjects with T1 and T3 tumors, respectively. The times to normalization for all three tumor-type groups were significantly different from one another.

There were no significant differences seen with respect to IMRT boost for PSA normalization < 1 ng/mL or for < 2 ng/mL (Tables 2 and 3). Similarly, there was no significant correlation seen within either of these two normalization groups between time to normalization and either age at time of treatment planning CT, length of hormone treatment, or pre-treatment PSA levels (pre-PSA). For the subjects with normalization of PSA to < 2 ng/mL, there was not a significant correlation between Gleason Score and time to normalization. However, there was a weakly significant correlation for those subjects with normalization of PSA < 1 ng/mL.

**Figure 1:**
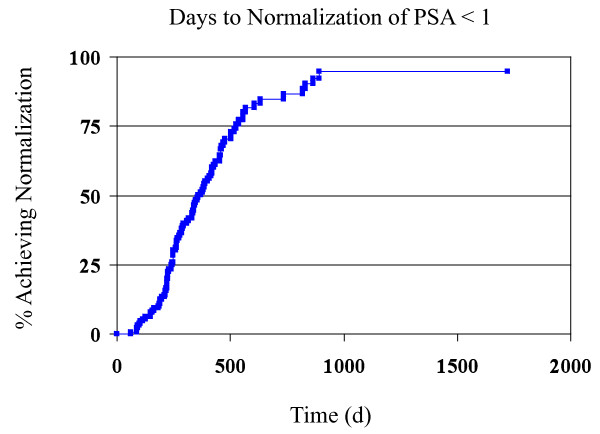
**Kaplan-Meier curve detailing the time for normalization of PSA to less than 1 ng/ml**.

**Figure 2:**
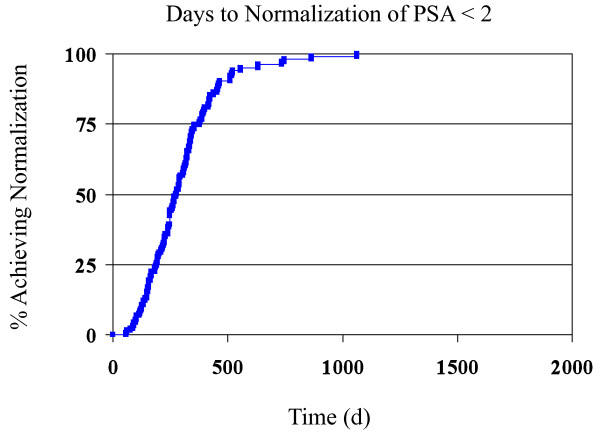
**Kaplan-Meier curve detailing the time for normalization of PSA to less than 2 ng/ml**.

Using the Kaplan-Meier method, times to PSA normalization were calculated, as shown in Figures 1 and 2. Multivariate analyses were performed using the three factors found to be statistically significant (for the purposes of this analysis, p < 0.2) for their effect on time to PSA normalization < 1 ng/mL: use of IMRT plus NAAD vs. IMRT alone, tumor stage T2, and Gleason score. The analysis demonstrated that all three variables were significant predictors of time to normalization. Hazard ratios with 95% confidence intervals were calculated for Stage 2 tumors (2.6; 1.4 - 4.8) and NAAD (0.5; 0.3 - 0.9). A separate analysis was performed for tumor stage T2 as a predictor for PSA normalization below 2 ng/mL. Tumor stage T2, as might be expected, showed a significant hazard ratio of 2.1 (95% CI 1.1 - 4.0).

## Discussion

The use of PSA as a tumor marker for prostate cancer is widespread and well-studied. The use of total serum PSA to identify patients with prostate cancer has been well-established since the early 1990 s and has resulted in a large increase in the detection of early stage prostate cancer [8]. Serum PSA is now widely used as a marker to determine responses to primary therapy, to monitor responses to hormonal therapy and to detect recurrent cancer.

PSA can be used to determine the efficacy of primary therapy including radical prostatectomy and radiation therapy. The PSA nadir after radical prostatectomy has been shown to correlate strongly with recurrence [9]. Patients treated with external beam radiation therapy quite often still have low but detectable levels of PSA upon completion of their therapy. Those who demonstrate three consecutive increases in PSA are considered to have recurred based upon the American Society of Therapeutic Radiology and Oncology's (ASTRO) definition [10]. Additionally, patients who experience a rise by 2 ng/mL or more above the nadir PSA after external radiation therapy with or without NAAD are considered to have biochemical failure according to the RTOG-ASTRO Phoenix definition [11].

Similarly, serum PSA can be utilized to determine response to hormonal therapy. Patients treated with androgen deprivation therapy often have dramatically reduced levels of PSA upon completion of their therapy. This decrease in PSA level has been shown to coincide with improved clinical symptoms in prostate cancer patients. Also, a PSA nadir of less than 0.4 ng/mL has been shown to be correlated with the duration of remission [12].

Our findings point to three distinct factors that appear to be involved in affecting the time to normalization of PSA after treatment for clinically localized prostate cancer with IMRT: tumor stage, Gleason score, and the use of NAAD therapy.

PSA levels normalized below both 2 ng/mL and 1 ng/mL at a much slower rate when the tumor being treated was a stage T2 tumor rather than when the tumor was stage T1 or T3. Patients with stage T2 tumors had an average time to normalization of 95 days longer than patients with stage T1 tumors and 221 days longer than patients with stage T3 tumors when normalizing to PSA < 2 ng/mL. Similarly, patients with stage T2 tumors had an average time to normalization to PSA < 1 ng/mL of 111 days longer than those with stage T1 tumors and 286 days longer than those with stage T3 tumors.

The difference in time to normalization of PSA < 2 ng/mL between stages T1, T2, and T3 was statistically significant. It could be hypothesized that those patients with stage T2 disease possess a different biologic phenotype that reacts more rapidly to androgen deprivation. It may be true that stage T3 cancers are more dispersed so as to allow a greater interaction between those malignant cells that make up the tumor and the circulating anti-androgen affects of the therapy. With a more extensive and richer connection to systemic blood supplies, the stage T3 tumor may also be more susceptible to the effects of the androgen deprivation therapy. Alternatively, potentially being more de-differentiated, T3 tumors may respond more rapidly to NAAD.

The T1 tumors also show a relative susceptibility to PSA normalization in comparison with T2 tumors. There is potentially a different explanation as to why they are more susceptible. Having less tumor burden, there is potentially a greater chance that T1 patients will undergo apoptotic cell death due to androgen deprivation to cause a significantly faster normalization of PSA level. The T2 tumors may have invaded to the point that a certain percentage of malignant cells will simply be untreated by the affects of androgen deprivation, but have not invaded to the point where their increased access to the systemic blood supply results in greater susceptibility to the anti-androgen affects of the therapy. Overall, it is difficult to make definitive conclusions regarding T stage and rate of PSA normalization, as only 5% of our sample had T3 disease and 15% had T2 disease.

Our data also showed a statistically significant correlation between the Gleason score of a tumor and the rate of PSA normalization to below 1 ng/mL, but not to below 2 ng/mL. This may possibly be explained by the fact that the cells that comprise a tumor with a high Gleason score are, by definition, less differentiated. It has been well documented that tumors of a higher Gleason score are made up of cells that actually produce less PSA per cell [13,14]. It then would follow that androgen deprivation therapy could very well have a more profound impact on the PSA production abilities of cells that were already less differentiated.

Finally, our data demonstrate that patients treated with IMRT plus NAAD normalized to a serum PSA level below 1 ng/mL 102 days earlier than those patients treated with IMRT alone. This effect was not significant when the level of PSA normalization was set at less than 2 ng/mL. It is possible that the use of NAAD therapy acts as a radiosensitizer in areas of the tumor mass. It is also possible that the androgen deprivation therapy is causing apoptotic cell death, as well as surviving tumor cells to cease production of PSA.

When androgen deprivation therapy is implemented, there is a subsequent apoptotic death of large numbers of cancerous prostate cells. This results in a significant decrease in the serum PSA level due to decreased prostatic cell mass. However, it has been shown that because transcription of the PSA gene is regulated by an androgen receptor, not all of this serum PSA decrease is due to cell death. Some surviving tumor cells are simply blocked from producing PSA because of the lack of androgen available to stimulate transcription of the PSA gene [15,16]. This phenomenon may also explain in part the significantly shorter time to PSA normalization when androgen deprivation therapy is combined with IMRT.

Serum PSA levels are routinely used today as a measure of a therapy's impact on prostate cancer. With such a significantly quicker normalization of PSA when neoadjuvant hormone therapy is used in conjunction with IMRT, this may constitute a reason to think more seriously about expanding the role of NAAD therapy in these patients. Further study is needed to elucidate whether the rate of PSA normalization is linked to notable endpoints such as mortality or disease recurrence. If it is found that a faster rate of PSA normalization to a level below 1 ng/mL is associated with decreased mortality or disease recurrence rates, then the use of NAAD therapy may need to be expanded to men with clinically localized disease.

Some work in this area has been done with some conflicting results. In a very large study that showed the importance of PSA normalization, Collette et al. assessed whether PSA could serve as a surrogate endpoint for survival [17]. They showed that, using a PSA normalization value of 4 ng/mL, those patients who normalized showed 4.9-fold greater odds of surviving than those patients who did not normalize. While this does not directly attest to the importance of the rate of PSA normalization, given the nearly 5-fold greater odds of survival for those patients achieving normalization, achieving that goal would be of significance.

Of additional concern is whether reduced time to PSA nadir is related to more positive outcomes. Chung et al. noted that, for PSA nadir values > 0.9 mg/mL, increased time to PSA nadir was associated with increased prostate cancer specific mortality and all causes mortality, relative to men with a time to PSA nadir < four months [18]. Conversely, both Ray et al. and Hori et al. have shown a direct relationship between positive outcomes and a decreased time to PSA nadir [19,20]. In our cohort, we did not correlate time to PSA normalization with clinical outcome.

Additionally, our data showed no significant relationship between the rate of normalization of PSA and the duration of NAAD therapy. Previous studies have attempted to determine the optimal duration of androgen deprivation therapy for men with clinically localized prostate cancer. One large study by Crook, et al. showed no difference in overall survival, disease-free survival, or rates of recurrence between two groups of men with clinically localized prostate cancer treated with either 3 or 8 months of neoadjuvant androgen deprivation therapy [21]. The same study, however, did show a difference in disease free survival among men with high risk disease. A number of other studies have also shown that, at least in men with high risk clinically localized disease, there may be significant benefits to a longer duration of androgen deprivation therapy [22,23]. Overall, studies have shown conflicting results in terms of whether the duration of androgen deprivation therapy has any true effect on mortality and disease recurrence rates.

## Conclusions

In summary, our results show that the use of neoadjuvant androgen deprivation therapy (NAAD) in conjunction with intensity modulated radiation therapy (IMRT) leads to a significantly shorter time to serum prostate specific antigen (PSA) normalization (less than 1 ng/mL) than the use of IMRT alone in the treatment of clinically localized prostate cancer. Also, factors leading to a shorter time to PSA normalization after IMRT treatment for clinically localized prostate cancer include tumor stages T1 and T3 and a higher tumor Gleason score.

## Competing interests

The authors declare that they have no competing interests.

## Authors' contributions

MDS obtained the retrospective data from the charts, and wrote the first draft of the paper. GDAP and PC conceived of the study, and participated in its design and coordination and helped to draft the manuscript. ATD ran the statistical analyses, and helped draft the manuscript. All authors read and approved the final manuscript.
